# A Synthetic European Weather Dataset Based on Spatiotemporal Vine Copulas

**DOI:** 10.1038/s41597-025-06015-3

**Published:** 2025-11-03

**Authors:** Judith N. Claassen, Elco E. Koks, Marleen C. de Ruiter, Philip J. Ward, Wiebke S. Jäger

**Affiliations:** 1 Institute for Environmental Studies, Vrije Universiteit Amsterdam, Amsterdam, The Netherlands; 2 Deltares, Delft, The Netherlands

**Keywords:** Climate sciences, Engineering

## Abstract

A stochastic weather generator provides data by capturing statistical properties of observed weather patterns, enabling the simulation of realistic time series beyond the historic record. Such simulated weather data can be valuable in many fields (e.g., agriculture and energy), where multiple variables (e.g., temperature and precipitation) influence the production processes. Here, we present a new European simulated dataset for temperature, precipitation and wind speed, generated by the MYRIAD-Stochastic vIne-copula Model (MYRIAD-SIM). MYRIAD-SIM captures both spatiotemporal and multivariate dependencies with the use of conditional vine copulas, a statistical tool. The statistical properties of the MYRIAD-SIM data closely resembles ERA5-Land data while maintaining sufficient variability to explore possible alternative scenarios. The simulated data can facilitate new insights in, for example, compound climate event research, by providing multivariate weather events across different conditions.

## Background & Summary

Stochastic weather data enable more comprehensive risk assessments in sectors such as finance, agriculture^1–3^ and energy^4,5^, by providing plausible weather events beyond the historical record. A commonly used tool for generating stochastic data of weather variables, such as temperature and precipitation, is stochastic weather generators (SWGs). SWGs produce realistic time series of a given length based on the statistical properties of historical observations^6^. Although the initial SWGs produced data for a single weather variable at a single location^7^, more recent SWGs are capable of producing multisite stochastic weather samples^8–12^. Multi-site stochastic weather data can be desirable, for example, to use spatial precipitation data to assess the probability of widespread meteorological drought^13^. Additionally, an increasing number of SWGs allow for generating stochastic data of multiple weather variables^9,10,12^. Multivariate stochastic data are particularly useful compared to single-variable data, as they can capture the complex dependencies between different weather variables, e.g. precipitation and temperature. This is crucial for applications, such as a drought assessment, where both precipitation deficits and high temperatures contribute to the severity of the drought^13^. To accurately model these dependencies, statistical methods are required. One widely used approach is copulas, which provide a flexible way to represent relationships between multiple variables^14^.

A copula is a multivariate statistical tool to describe the joint probability between variables, while allowing each variable to have their own individual marginal distribution, making it suitable for modeling non-linear dependence^14,15^. Copulas can also be used to model the dependence between multiple variables, for example, between compound hot, dry and windy conditions^16^, or to understand the impact of compound hot and dry conditions on vegetation^17–19^. Here, either a multivariate copula or a vine copula is applied. Multivariate copulas assume that the dependence between all variables is the same, and vine copulas decompose the dependence structure into separate bivariate copulas. Although multivariate copulas often require fewer parameters and are more computationally efficient, they are less flexible compared to vine copulas, which can better capture the complex dependency between variables^18,20^. Vine copulas are also suitable for sampling random and conditional samples of variables to better estimate joint probabilities and generate stochastic events. For example, Eilander *et al*. generate thousands of random samples of rainfall, surge, discharge, and precipitation to study compound flooding in Mozambique^21^.

However, such copula-based simulations are often limited to samples at a specific location at a specific time, not considering the spatiotemporal dependencies between variables, which are crucial for accurately modeling widespread weather phenomena. To address this, some stochastic weather generators have incorporated copulas to model dependencies between time steps^22,23^ and at different locations^8,11^. However, these SWGs are specific to one variable, i.e. precipitation, and tailored to specific sites in specific case study areas. Despite these limitations, these models have illustrated that copulas are a suitable tool to model spatiotemporal dependence, while previous studies have already shown their effectiveness in modeling dependencies between different weather variables. This suggests that copulas have the potential to jointly model both aspects within an integrated model.

Based on the demonstrated ability of copulas to model both spatiotemporal dependencies in SWGs and dependencies across variables, this paper presents a fully copula-based SWG for Europe, named the MYRIAD-Stochastic vIne-copula Model (MYRIAD-SIM). Using the flexibility of vine copulas, MYRIAD-SIM generates realistic simulations that preserve complex dependencies across space, time, and climate variables, namely temperature, precipitation, and wind speed, at a European scale. The simulated output data of the model will enable new insights into compound climate events by providing stochastic data to study the frequency and magnitude of co-occurring extreme events, across various locations and time scales.

## Methods

In this paper, we produce a stochastic dataset of daily temperature, precipitation, and wind speed on a European scale, using MYRIAD-SIM. The Model is fit to multiple variables that are on the same spatiotemporal scale, so that the simulated data that the model produces preserve the dependency between the variables, for example, the dependency between wind speeds and precipitation. The following subsections describe the process of fitting the model to the data. First, an introduction to the essential copula theory underlying the model is provided. This is followed by a description of the dataset used for training, an explanation of the model fitting procedure, and a presentation of the stochastic data generation algorithm.

### Essential Copula Theory

#### Bivariate copulas

MYRIAD-SIM is based on the statistical properties of the data using bivariate and vine copulas. A copula is a method used to describe the dependence structure between several random variables and can model the joint distribution of multiple variables separately from their marginal distributions. Therefore, the copula is a multivariate distribution with all univariate margins being uniform on [0, 1]^24^. Sklar’s Theorem states that any multivariate joint distribution function can be decomposed into marginal distributions and a copula function that describes the dependence structure between the variables^25^.

**Sklar’s Theorem**: Let *F* be the joint distribution function of a multivariate random vector (*X*_1_, *X*_2_, …, *X*_*n*_), with marginal distribution functions *F*_1_, *F*_2_, …, *F*_*n*_ for each variable. Then, there exists a copula function *C*: [0, 1]^*n*^ → [0, 1] such that for all (*x*_1_, *x*_2_, …, *x*_*n*_) in the support of *F*, we have:

1 $$F({x}_{1},{x}_{2},\ldots ,{x}_{n})=C({F}_{1}({x}_{1}),{F}_{2}({x}_{2}),\ldots ,{F}_{n}({x}_{n})),\,{\bf{x}}\in {{\mathbb{R}}}^{n}$$

To fit a copula to the data, the data must first be transformed into uniform margins. This is achieved via the Probability Integral Transform (PIT). PIT states that if *X* is a continuous random variable with cumulative distribution function (CDF) *F*_*X*_(*x*), then the transformed variable *u* = *F*_*X*_(*x*) has a uniform distribution on the interval [0, 1].

Once the data have been transformed into their uniform margins, different copulas can be compared to find the best fit. The copulas listed in Table 1 have been used in the model described in this paper. These copulas were selected because their inverse conditional CDFs have an analytical solution. Hence, they do not need to be computed numerically. Inverse conditional CDF is often required to sample from a copula; therefore, being able to solve it analytically significantly reduces the computation time.

**Table 1:** Selected copulas and their functional expressions.

Copula	C(u_1_, u_2_)	Parameters
Gaussian	Φ_*θ*_(Φ^−1^(*u*_1_), Φ^−1^(*u*_2_))	*θ* ∈ [−1, 1]
Frank	$-\frac{1}{\theta }\log \left[1+\frac{(\exp (-\theta {u}_{1})-1)(\exp (-\theta {u}_{2})-1)}{\exp (-\theta )-1}\right]$	$\theta \in {\mathbb{R}}\backslash \{0\}$
Clayton	${\left[\max \left\{{u}_{1}^{-\theta }+{u}_{2}^{-\theta }-1;0\right\}\right]}^{-1/\theta }$	*θ* ∈ [−1, *∞*)⧹{0}
Clayton 90 degrees	${u}_{2}-{\left[\max \left\{{(1-{u}_{1})}^{-\theta }+{u}_{2}^{-\theta }-1;0\right\}\right]}^{-1/\theta }$	*θ* ∈ [−1, *∞*)⧹{0}
Clayton 180 degrees	${u}_{1}+{u}_{2}-1+{\left[\max \left\{{(1-{u}_{1})}^{-\theta }+{(1-{u}_{2})}^{-\theta }-1;0\right\}\right]}^{-1/\theta }$	*θ* ∈ [−1, *∞*)⧹{0}
Clayton 270 degrees	${u}_{2}-{\left[\max \left\{{u}_{1}^{-\theta }+{(1-{u}_{2})}^{-\theta }-1;0\right\}\right]}^{-1/\theta }$	*θ* ∈ [−1, *∞*)⧹{0}

#### Vinecopulas

**Fig. 1:**
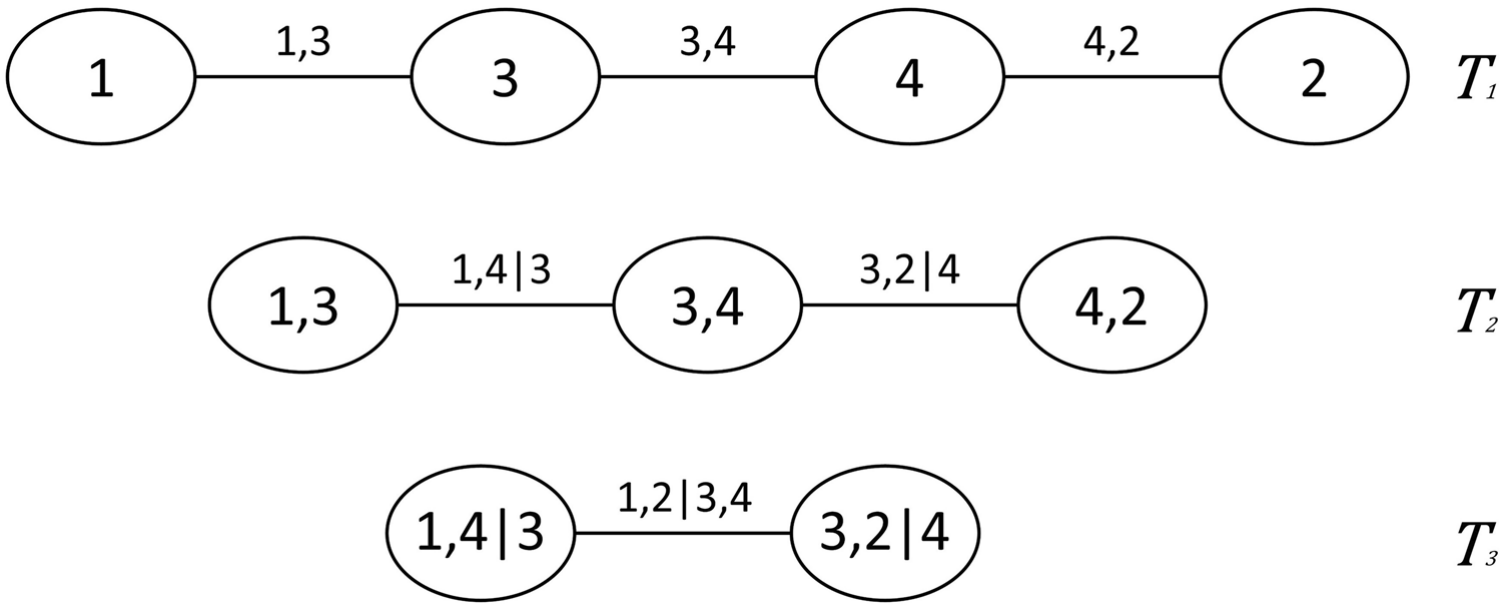
Example of a vine tree structure of a vine copula. Many other structures are also possible.

A vine copula model is a method to construct multivariate copulas with the use of bivariate copulas as building blocks. We follow the theory provided by Czado *et al*.^14^. The basis of a vine copula is conditioning^14,26^. A nested sequence of trees can be used to describe a vine copula. Each tree consists of nodes and edges, where nodes represent variables, and edges represent the bivariate copulas (dependencies) between them, as illustrated in Fig. 1.

For a *d*-dimensional vine distribution, the first tree, *T*_1_, identifies *d* − 1 pairs of variables (edges), which are associated with a bivariate copula. This is shown in Fig. 1, where there are four variables (*d* = 4), and there are three pairs. The second tree, *T*_2_, identifies *d* − 2 pairs of variables, whose distribution, conditional on a single variable, is also modeled by a pair-copula. In *T*_2_, the first conditioning variables are determined, which in Fig. 1 are variables 4 and 3. The subsequent trees will have *d* − *i* pairs, where *i* is the tree number, until there is only one pair left. The final conditioning set in *T*_*d*−1_ consists of all the conditioning variables in the preceding trees. In Fig. 1, it can also be observed that the edges of one tree become the nodes of the next. Two nodes in *T*_*i*+1_ are only connected by an edge if these nodes share a common node in tree *T*_*i*_.

A vine copula can be fitted by testing all the different possible vine structures and minimizing the model selection criteria, such as the Bayesian Information Criterion (BIC) and the Akaike Information Criterion (AIC). This method is feasible when there are only a small number of variables. For example, there are only three possible vine structures for a vine copula of three variables. However, as the number of variables increases, the number of possible vine structures increases super-exponentially as $d!\times {2}^{\frac{(d-2)(d-3)}{2}-1}$^27^. This means that with, for example, six variables, there are already more than 20,000 possible vine structures. Therefore, the Dißmann fitting algorithm^28^ has been developed, which fits a vine copula by fitting the strongest dependencies first and selecting appropriate bivariate copulas using criteria such as AIC or BIC.

Dißmann *et al*. also developed a sampling algorithm to draw random samples from a vine copula^28^. In this algorithm, the samples are drawn in a specific sampling order. The sampling order is defined by the structure of the copula tree. For each vine copula with *d* variables, there are 2^*d*−1^ implied sampling orders^29^. For example, the vine copula in Fig. 1 has four variables, therefore there are eight possible sampling orders. The sampling order of a vine copula is important when samples need to be conditioned on a single variable or set of variables. In order to generate conditioned samples, the conditioned variable is required to be at the end of the sampling order.

For a given copula structure, the desired sampling order may not exist. In such cases, the copula must be refitted to ensure that the desired sampling order is achieved. Since the model described in this paper requires a specific conditioning of certain variables, an adapted Dißmann fitting algorithm is used to identify the copula structure that aligns with the desired sampling order^30,31^. Conditioned samples are also drawn using an adapted Dißmann fitting algorithm by Claassen *et al*.^30,31^. The algorithms for fitting and sampling the vine copulas are implemented in the *VineCopulas* Python package^30,31^, which has been utilized in the model described in this paper.

There are different possible types of vine structure, more commonly known as the C-vine, R-vine and D-vine^14^. However, in this model we only considered the D-vine structure. A D-vine structure, as seen in Fig. 1, consists of only trees that are single paths. This means that all nodes, with the exception of two, have a neighbor on each side of it. In a D-vine sequence, *T*_1_ already determines all pairs in the subsequent trees. Therefore, to define a D-vine structure, only the path in *T*_1_ needs to be specified, also referred to as the order of a D-vine. Since only *T*_1_ needs to be defined to establish a specific sampling order, the D-vine is a suitable choice for this model, as it significantly improves computational efficiency by reducing the possible number of vine structures.

### Data

To develop the MYRIAD-SIM dataset, the model is fitted using climate variables from the ECMWF ERA5-land reanalysis dataset^32^. The variables used are shown in Table 2.

**Table 2:** Overview of the original data characteristics prior to preprocessing.

Name	Units	Spatial Resolution	Temporal Resolution	Temporal Coverage
10m u-component of wind	ms^-1^	0.1^°^ × 0.1^°^	Hourly	1950 to present
10m v-component of wind	ms^-1^	0.1^°^ × 0.1^°^	Hourly	1950 to present
2m temperature	K	0.1^°^ × 0.1^°^	Hourly	1950 to present
Total precipitation	m	0.1^°^ × 0.1^°^	Hourly	1950 to present

The original data listed in Table 2 have been downloaded from 2008 to 2023 for Europe, to obtain indicators for temperature, wind speed, and precipitation. This 16-year period is used to represent the recent climate, providing the most current observational data and capturing relevant seasonal and interannual variability, as well as several extremes. The temporal resolution of each variable was reduced to daily by calculating the daily mean for temperature, the u-component of the wind, the v-component of the wind, and the daily sum for precipitation. The unit of temperature has been converted from Kelvin to Celsius by subtracting 273.15. The wind speed of the components u and v has been calculated as follows:

2 $$\,{\rm{W}}{\rm{i}}{\rm{n}}{\rm{d}}\,{\rm{s}}{\rm{p}}{\rm{e}}{\rm{e}}{\rm{d}}\,=\sqrt{{u}^{2}+{v}^{2}}$$

### Model setup

#### Fitting

**Fig. 2:**
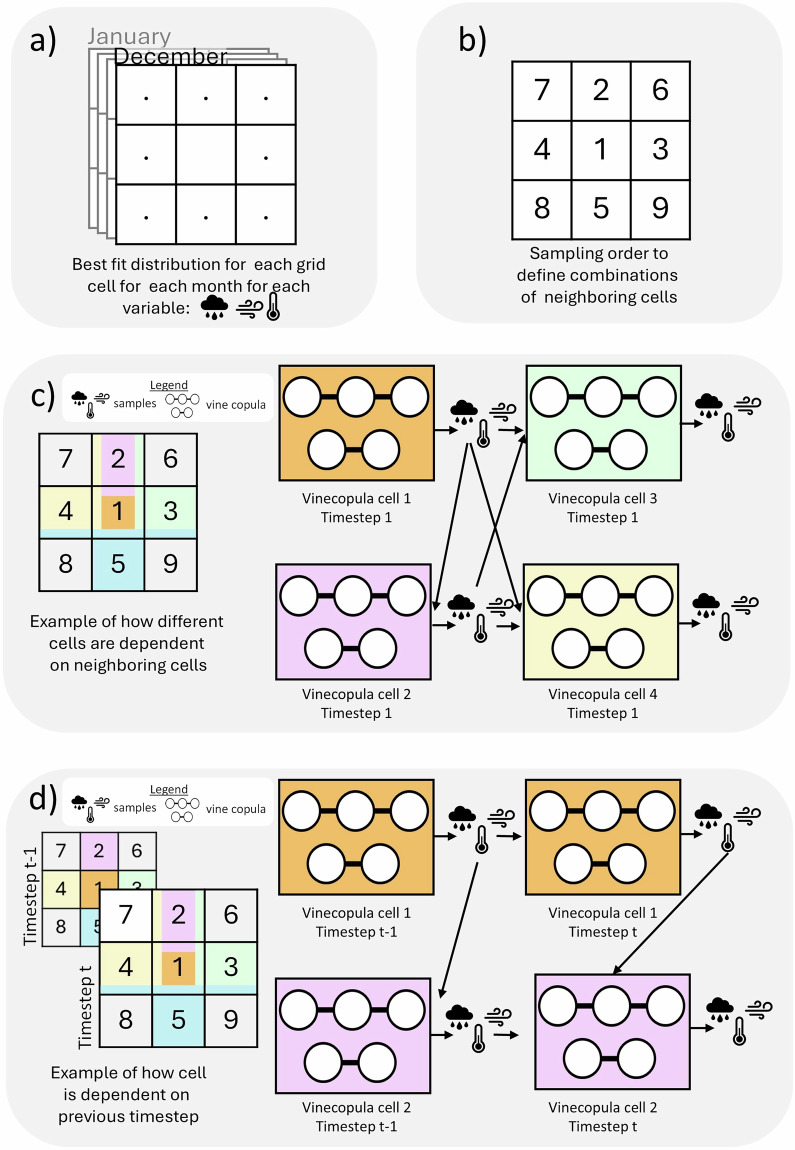
Schematic overview of the model setup. (**a**) Best fit marginal distribution selected for each variable, grid cell and month. (**b**) Grid cells are numbered from 1 to *N*, to define sampling sequence. (**c**) The model sequentially samples each grid cell based on previously sampled neighboring cells using vine copulas. Here, the colors in the cells to the left refer to those shown in the vine copula to the right. When a color appears across multiple cells, the cell with that color at its center is the one being sampled, conditioned on the cells where that color appears as a stripe. A cell may contain multiple colors, indicating that multiple cells are dependent on it. (**d**) The temporal relationship is incorporated by extending the sampling sequence to include each cell’s value from the previous time step, ensuring dependencies across both space and time are captured using copulas.

As explained previously, to fit a copula to the data, the data must first be transformed into uniform margins (see Fig. 2a). To obtain the CDF, the best-fit marginal distribution is selected for each grid cell based on the mean squared error using the fitting algorithm in the *VineCopulas* package^31^. This fits the best marginal distributions of individual variables using the univariate distributions available in the statistical Python package *SciPy*^33^. Furthermore, to simulate seasonal patterns, a distribution is fitted for each month of the year separately.

Once the distributions have been fit and the data transformed into uniform margins, each grid cell is assigned a number from 1 to *N*, where *N* is the total number of grid cells. Here, the grid cell at the center of the grid is assigned the number 1, and cells are numbered outward, with those closest to the center numbered first, and those furthest away numbered last. The distance is defined as the sum of the absolute differences in the row and column indices. Therefore, diagonal neighbors are considered farther away than horizontal or vertical neighbors. Among cells that share the same distance, numbering proceeds in clockwise order, starting with the cell directly above the center. This procedure defines the sampling sequence, used after the model is fitted, with cell 1 sampled first and cell *N* sampled last. An example of the numbering can be seen in Fig. 2b.

We model three types of dependence: spatial dependence, temporal dependence, and dependence between different variables. To capture these relationships at each grid cell, we use two vine copulas. The first vine copula captures dependence in space and between variables (Fig. 2c), while the second captures dependence across space, time, and variables (Fig. 2d). In this section, we refer to temperature, precipitation, and wind speed as $t2{m}_{n}^{t}$, $p{r}_{n}^{t}$ and $w{s}_{n}^{t}$ respectively, where *t* indicates the time step and *n* the grid cell number, which ranges from 1 to *N*. In this model, each cell is sampled based on the values of its neighboring grid cells that have been sampled earlier.

In the example in Fig. 2c, the step that captures the spatial dependence at *t* = 1 is illustrated, where cell 1 is sampled first. Therefore, cell 1 is not dependent on neighboring grid cells, and the samples for $t2{m}_{1}^{t}$, $p{r}_{1}^{t}$ and $w{s}_{1}^{t}$ (i.e. temperature, precipitation, and wind speed) at time step *t* = 1 are drawn from the copula fit between only these three variables in cell 1. This cell and vine copula are indicated by the orange shading in Fig. 2c. Next, cell 2 is sampled based on cell 1, therefore, a copula fit between the variables of cell 1 and 2 is used to sample $t2{m}_{2}^{t}$, $p{r}_{2}^{t}$ and $w{s}_{2}^{t}$ conditioned on samples $t2{m}_{1}^{t}$, $p{r}_{1}^{t}$ and $w{s}_{1}^{t}$. This is also illustrated in Fig. 2c, where the samples from cell 1 (shown in orange) are used to sample cell 2 (shown in pink). Similarly, cell 3 (shown in green) is sampled based on the values of cells 1 and 2, which requires a vine copula to model the relationship between nine variables ($t2{m}_{n}^{t}$, $p{r}_{n}^{t}$ and $w{s}_{n}^{t}$ for cells 1, 2, and 3) conditioning on the variables in cells 1 and 2. This approach is generalized for all grid cells, fitting vine copulas to describe the relationships between directly neighboring cells sampled before the cell of interest, as is shown on the right-hand side of Fig. 2c. This ensures that spatial dependencies between grid cells are preserved.

**Fig. 3:**
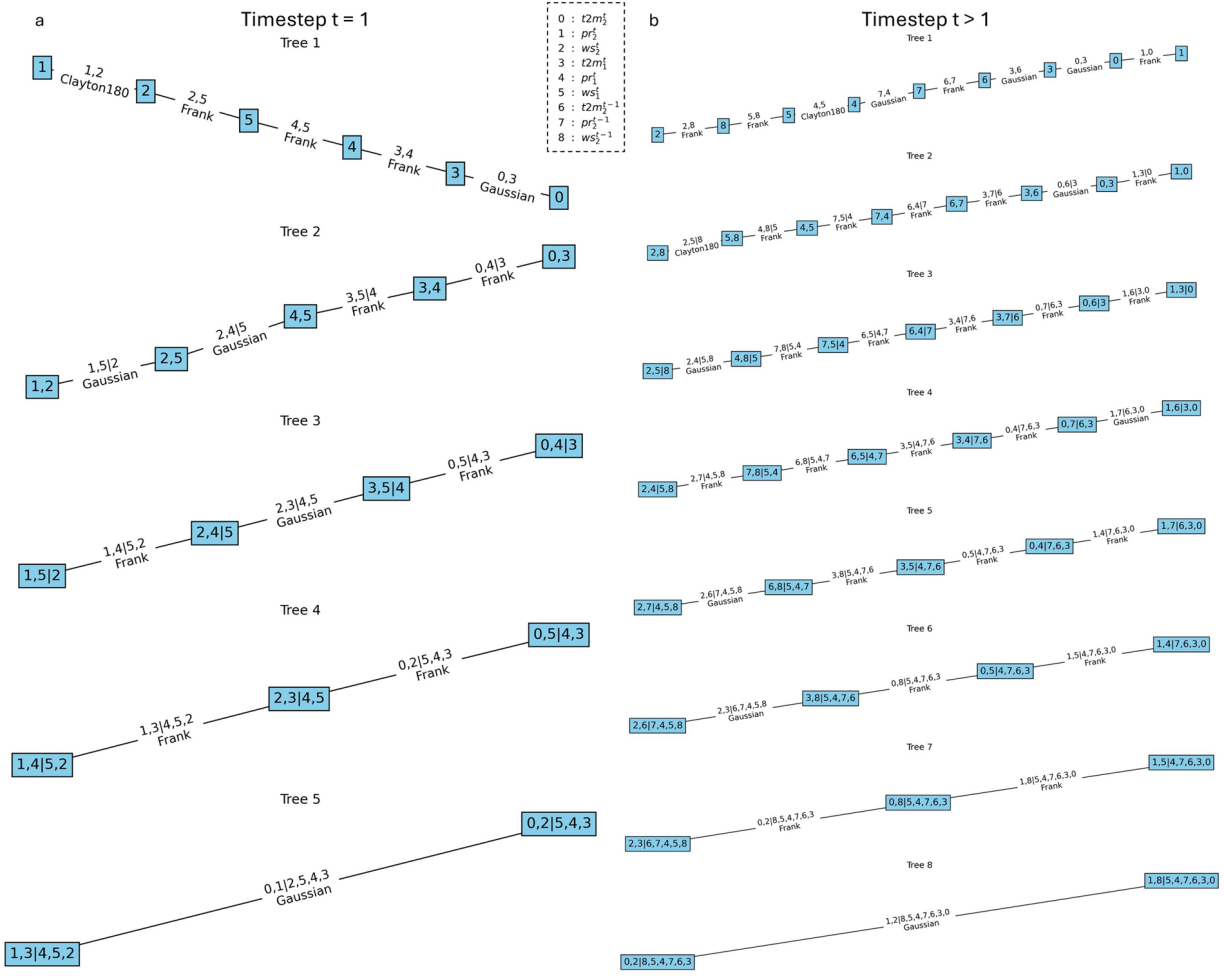
Vine copula structure for *n* = 2. (**a**) Vine copula when *t* = 1. (**b**) Vine copula when *t* > 1.

To incorporate the temporal relationship into the model, the same sampling sequence is used in space. However, in addition to neighboring grid cells, each cell’s value from the previous time step (*t* − 1) is also considered, as shown in Fig. 2d. For example, cell 1 at the current time step (*t*) can be sampled based on cell 1 from the previous time step (*t* − 1), fitting a copula between the values of cell 1 at *t* and *t* − 1. Similarly, cell 2 at *t* can be sampled based on the values of cell 1 at *t* and cell 2 at *t* − 1. This is illustrated in Fig. 2d, where cell 1 and cell 2 are again shown in orange and pink, respectively. Figure 3 presents an example of the vine copulas for grid cell *n* = 2, illustrating a real vine copula structure from the model and highlighting the differences between the vine copula at *t* = 1 and *t* > 1. Each cell in the grid has a unique vine copula structure. Some of these structures are more complex than the example shown in Fig. 3. For example, cell 5 in Fig. 2 depends on three previously sampled cells, namely: cells 1, 3, and 4. For each additional neighboring cell, three nodes (one per variable) are added to the vine structure. This results in 12 nodes for cell 5 in the first time step and 15 nodes in subsequent time steps, when the previous time step of cell 5 is also included. Cells like cell 5, which have three direct neighbors, represent the most complex cases included in the model.

Similarly to the best-fit distributions of each variable in each grid cell, the vine copulas are also fit for each grid cell for each month to capture the seasonality. Additionally, if there is no direct neighbor sampled previously, the copula is fit between the cell of interest and the nearest cell available. This could be the case, for example, when there is a sea or a lake.

#### Generating samples

To generate simulated samples, the model is run time step by time step. In the first time step, the fitted copulas and vine copulas for the different grid cells with respect to neighboring grid cells are used to sample. In the following time steps the vine copulas that were fit on the neighboring grid cells and previous grid cells are used. While the model’s spatiotemporal dependencies are technically limited to immediate neighboring grid cells and the previous time step, the conditional sampling method constrains the possible outcomes, resulting in spatiotemporally coherent simulated data. Furthermore, in order to decrease the running time of the model, it is fit and run on a coarse grid. Here, the resolution of the grid has been reduced by a factor of 10 (from 0. 1^°^ to 1^°^), by averaging the higher resolution grid cell. The resulting grid has a dimension of 44 rows by 61 columns, of which 1076 cells correspond to land areas where data are available. This change in resolution significantly reduces both the time required to fit the model and the number of samples that need to be drawn at each time step. The simulated data consist of sixteen ensemble members of sixteen years. Each ensemble member runs from 2008 to 2023, which is the same time span as the training data.

## Data Records

The MYRIAD-SIM data are publicly available on Zenodo^34^. The data for temperature, precipitation and wind speed are stored in a separate NetCDF file for each variable. Each NetCDF file is approximately 1 GB in size and contains four dimensions and five attributes, as described in Table 3. The data variable names are t2m, pr, ws, for temperature, precipitation and wind speed respectively.

**Table 3:** Description of dimensions and attributes in the dataset NetCDF files.

Category	Description
**Dimensions**	
ens	Ensemble member index (0 to 15), representing different model runs.
time	Time dimension, ranging from 2008-01-01 to 2023-12-31.
latitude	Latitude values (degrees north).
longitude	Longitude values (degrees east).
**Attributes**	
units	Units of measurement for each variable.
standard_name	Full name of each variable.
description	Brief explanation of each variable.
version	Version number of the dataset.
created	Date when the dataset was generated.

## Technical Validation

As The MYRIAD-SIM dataset is based on ERA5-land, we validate our model results by comparing the two. The validation will evaluate summary statistics, seasonality, spatial and temporal duration of extremes, and dependence between variables.

**Fig. 4:**
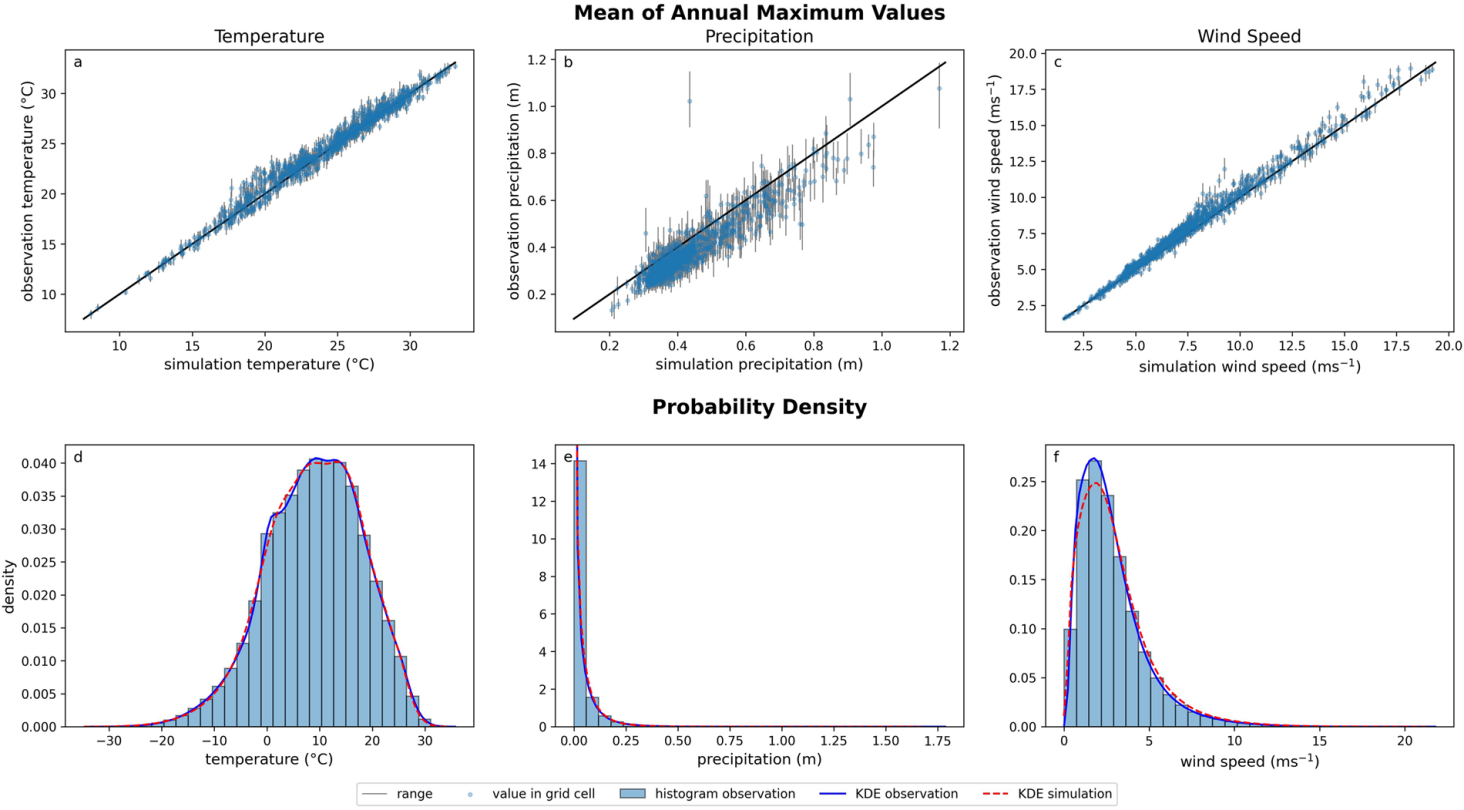
**a**–**c**) Mean of the annual maximum values for the observations and simulations. Each point represents a grid cell and the grey bars represent range between different ensemble members. (**d**–**f**) Histogram and kernel density estimation (KDE) of observed data, and KDE of simulated data, for all grid cells andtime steps.

Figure 4 shows the mean annual maximum values of the three variables, temperature, precipitation, and wind speed. In general, the model appears to accurately simulate each variable. The maximum temperature is overestimated with a bias of 0.26 ^°^C. Here, bias is defined as the average difference between the observed and simulated values. The temperature shows little variability between the different members of the simulated ensemble. Precipitation is underestimated with a bias of -0.05 m. Precipitation shows the greatest variability between the members of the ensemble. The wind speed shows a positive bias of 0.29 ms^-1^. The kernel density estimation (KDE), shows that the data distribution of temperature and precipitation are well fit. The wind speed KDE of the simulation is more skewed to the right compared to the observation. Therefore, the simulated data may underestimate the frequency of moderate values, while overestimating the occurrence of higher values. To further evaluate this, we used the Wasserstein distance, which measures the difference between two probability distributions by quantifying the cost of transforming one distribution into the other^35^. The Wasserstein distance between the observed and simulated KDE is 0.15. This distance can be evaluated relative to the data range. For the wind speed, which ranges from 0 to approximately 20 m s^-1^, the Wasserstein distance corresponds to approximately 1% of the total range. This low percentage suggests that the simulated KDE is sufficiently accurate.

**Fig. 5:**
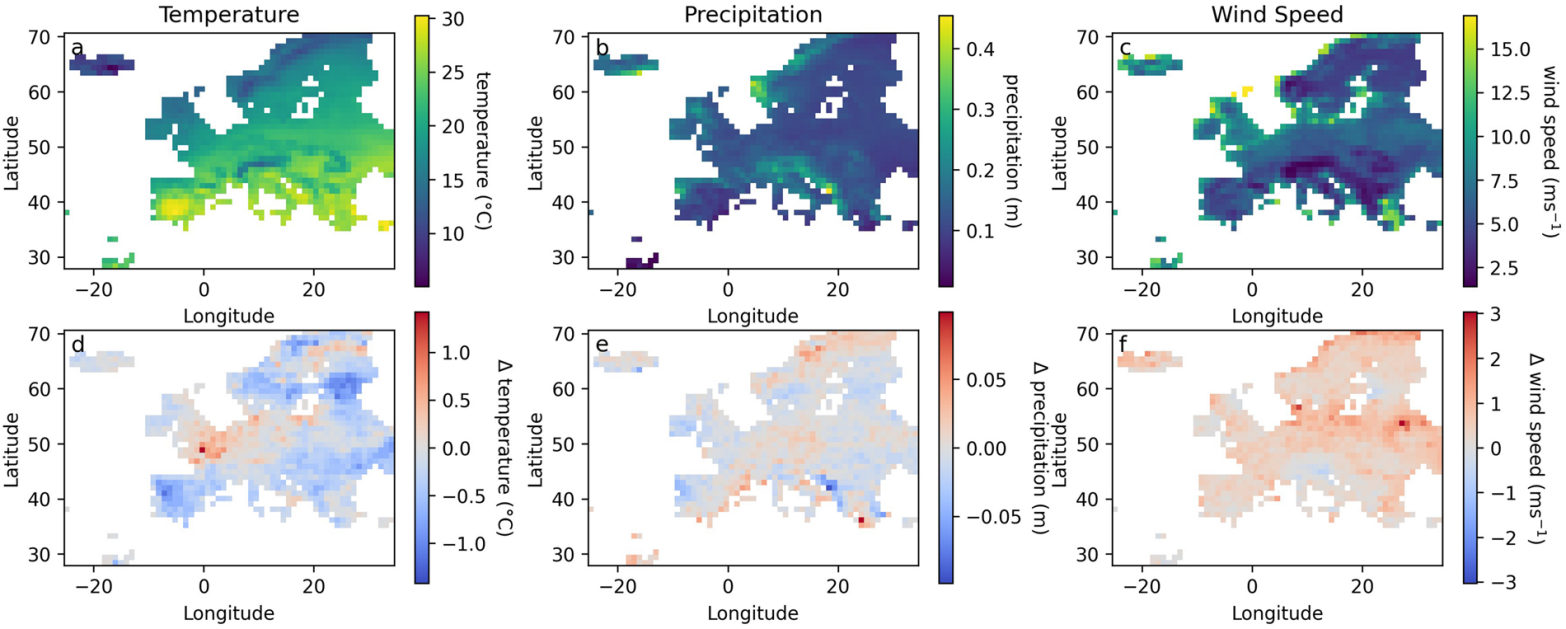
**a,b**) 95^th^ percentile of observed temperature and precipitation. (**c**) 99^th^ percentile of observed wind speed. (**d**–**f**) Bias between the percentile of observed vs. simulated data, where red colors show positive bias and blue colors indicate a negative bias.

Figure 5 shows the 95^th^ percentile of observed temperature and precipitation, and the 99^th^ percentile of wind speed as well as the bias of the respective percentiles between observed and simulated data. For wind speed the 99^th^ percentile was selected as this is more commonly used in wind hazard assessments to evaluate the most extreme events^36,37^. The temperature percentile shows a low bias, with most grid cells having a bias below 1 degree Celsius. There is a notably larger positive bias between the UK and France, and a larger negative bias in parts of Spain and Portugal. However, these biases are still below 2 degrees. The bias for the precipitation percentile also shows both negative and positive biases. These biases show no more than a 0.07 m difference between the observed and simulated data. The bias for wind speed is predominantly positive, which could also be observed in Fig. 4. The highest bias of approximately 3 ms^-1^ is also observed in areas with the highest 99^th^ percentile, such as the coast of Norway and Denmark.

**Fig. 6:**
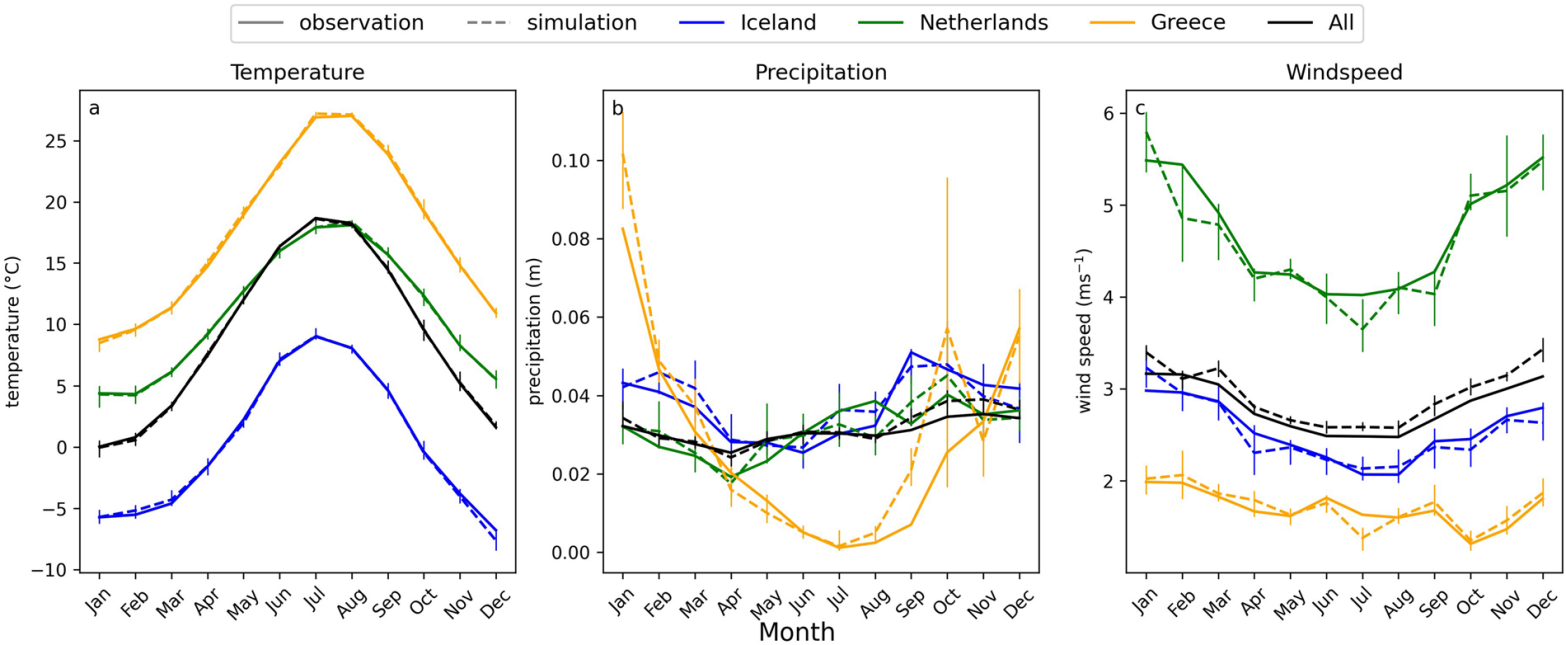
The monthly mean of each variable for all of Europe and at different locations for each variable. The solid line shows the observed data, the dashed line shows the simulated data, and the bars show the range between different ensemble members.

To evaluate the seasonality and spatial patterns of the simulated data in comparison to the observed data, the monthly mean at different locations is plotted for each variable, as well as the monthly mean aggregated for all locations (Fig. 6). Europe is located in the northern hemisphere and generally has four seasons with Winter in December-February and Summer in June-August. This seasonal pattern is also visible in Fig. 6a, where the black line indicates the monthly mean temperature throughout Europe, showing the highest values in summer and the lowest values in winter. However, depending on latitude, proximity to water, and altitude, seasonal patterns can vary. For example, the mean temperatures in Iceland (blue) are much lower compared to Greece (yellow). In general, there is good agreement between observation and simulation for the monthly mean temperature values in all regions, which captures seasonality well. Precipitation shows more varying patterns compared to temperature between different regions. For example, Greece has a wet season in winter and a dry season in summer. This seasonality is captured in the simulated data, with notably higher precipitation levels in January, February, September, and October than observed. Iceland and the Netherlands both experience precipitation year-round. The simulated precipitation in Iceland and The Netherlands fall within the margins of the simulated ensemble member in each month. Wind speed also shows a general seasonality, with higher average wind speeds in winter compared to summer. The highest wind speeds in this figure are observed in the Netherlands. While, the simulated data tends to overestimate the wind speed with a positive bias, in the Netherlands, the mean monthly wind speeds are underestimated. However, for most months, the monthly average of the observed wind speed remains within the range of the simulated ensemble members.

**Fig. 7:**
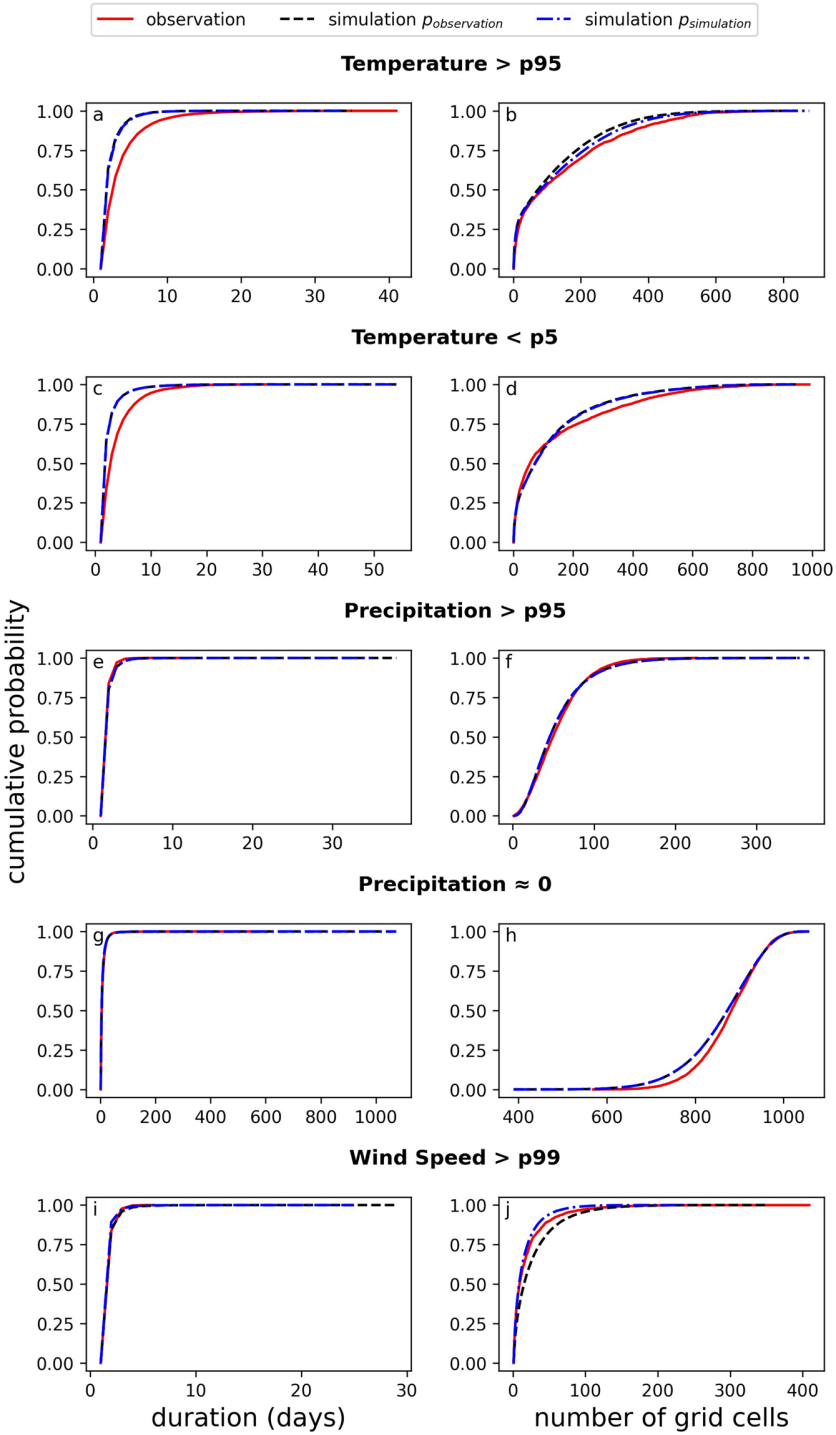
Cumulative probability of duration and number of grid cells above or below a certain percentile or value. Duration is calculated as the number of consecutive days a grid cell is above or below a certain value. number of grid cells is calculated as the number of grid cells that are above or below a certain value on the same day. The red line is the cumulative probability of the observation. The black dashed line is de cumulative probability of the simulation with respect to the percentiles of the observation. The blue dashed line is de cumulative probability of the simulation with respect to the percentiles of the simulation.

As MYRIAD-SIM incorporates both spatial and temporal dependencies, the simulated data should adequately capture the size and duration of extreme events. Figure 7 shows the cumulative probability of duration and size of different extremes. This includes both high extremes, such as hot temperatures, as well as low extremes, such as cold temperatures, where relevant. Figure 7a evaluates the duration of extreme high temperatures, defined as the number of consecutive days on which the temperature is above the grid cell’s 95^th^ percentile. The likelihood that there are approximately zero to ten days of high temperature is higher in the observations compared to the simulation. The maximum duration in the observation is similar to those in the simulation, around forty days. Figure 7b shows the cumulative probability that the temperature in a certain number of cells in Europe is above the 95^th^ percentile. Here, the observed and simulated data show very similar results, which may indicate that the spatial relationships are captured well. Figure 7c,d show similar results compared to Fig. 7a,b, however, here extreme low temperatures are evaluated where the values of the grid cells are below the 5^th^ percentile. However, the cumulative probability in 7d deviates between observation and simulation, insinuating that there is a higher probability of having between 150 and 600 grid cells experiencing cold conditions in the simulation. Figure 7e–h show the cumulative probability for duration and size of extreme high (above 95^th^ percentile) and low (values that are 0 when rounded to two decimals) precipitation. The cumulative probability of both high and low precipitation for the duration is similar for both the observation and the simulation. In the simulation, there are occurrences of longer wet and dry periods compared to the observation. However, these occurrences have a very low probability. Likewise, there is a possibility that a larger number of grid cells is above the 95^th^ percentile in the simulated data, although this also has a low likelihood. The cumulative probability that a specific number of grid cells is experiencing approximately zero precipitation appears very similar for both the observation and simulation. Finally, 7i,j show the cumulative probability of the duration of extreme wind speed (above 99^th^ percentile). Like the precipitation, the simulations show potential for longer durations of extreme wind in the simulated data vs. the observed data. While having extreme wind for over 30 days seems unlikely, it should be noted that ‘extreme’ in this case is relative to the location’s percentiles. As seen in Fig. 5c, the 99^th^ percentile of wind speed inland can be relatively low, and 30 consecutive days of wind speed above the 99^th^ percentile would not insinuate 30 days of stormy conditions. This also applies to long durations of high precipitation. Figure 7j shows the cumulative distribution of the number of grid cells with high wind speed at the same time. Here, it can be observed that there is a higher likelihood that there are between zero and 100 grid cells above the 99^th^ percentile in the simulated data. This is likely due to the positive bias in wind speed in the simulation, as was shown in Figs. 5 and 4. Therefore, when the number of grid cells are counted with respect to the 99^th^ percentile of the simulated data, the cumulative probability is much more similar between the simulated and observed data, indicating that the vine copulas do capture the general relationship well, and the bias likely originates from the marginal distribution.

**Fig. 8:**
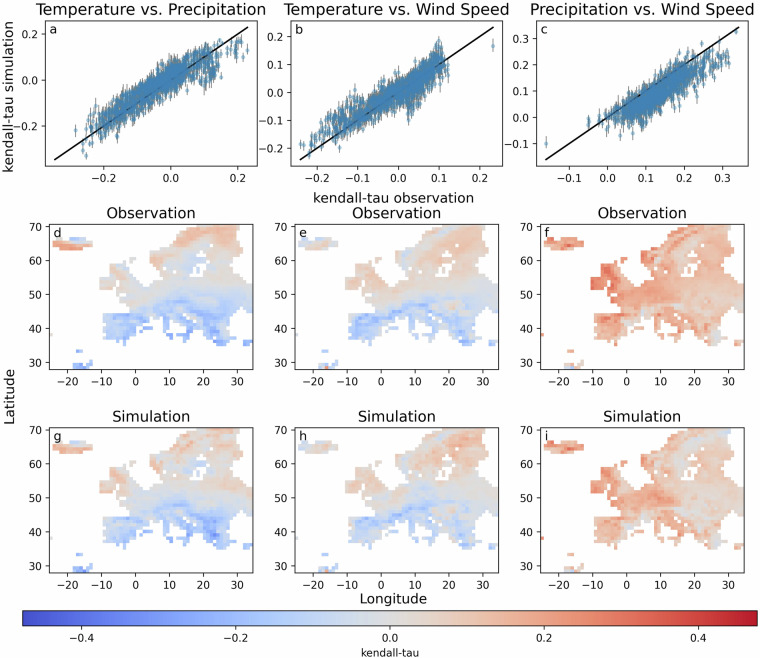
**a**–**c**) Kendall-tau between different variable pairs for the observations and simulations. Each point represents a grid cell and the grey bars represent range between different ensemble members. (**d**–**f**) Kendall-tau between different variables for different grid cells in the observed data. (**g**–**i**) Kendall-tau between different variables for different grid cells in the simulated data.

Finally, as MYRIAD-SIM data aim to capture the dependencies between temperature, precipitation, and wind speed, Kendall rank correlation coefficient (Kendall-tau) is used to evaluate the correlation between the variables. The Kendall-tau coefficient ranges from -1 to 1, where a positive value indicates that as one variable increases, the other tends to increase too, while a negative value suggests that as one variable increases, the other tends to decrease. Figure 8 shows the Kendall-tau between the three variable pairs, where each column of the figure evaluates a different pair of variables, temperature vs. precipitation, temperature vs. wind speed, and precipitation vs. wind speed. The Kendall-tau between temperature and precipitation can be both negative and positive as can be observed in Fig. 8a,d,g. Interestingly, Fig. 8d shows that the Kendall-tau is often positive in the north of Europe and negative in the south of Europe. This pattern also appears to be captured in the simulated data. Figure 8a shows that occasionally the simulated data has a negative Kendall-tau instead of a positive Kendall-tau, or a positive Kendall-tau instead of a negative one. However, since the Kendall-tau is often close to zero, these changes are not significant. The Kendall-tau between temperature and wind speed (Fig. 8b,e,h) show patterns similar to those between temperature and precipitation, with more frequent positive Kendall-tau in the north and negative Kendall-tau in the south. Here, the simulated data also capture the patterns well, including, for example, the negative Kendall-tau patterns in Scandinavia. The correlation between precipitation and wind speed is the highest, showing almost only positive Kendall-tau values. Although the simulated data appear to capture the relationship well, as can be seen in Fig. 8c,f,i, the Kendall-tau in the simulated data is often lower. However, most of the areas where the absolute Kendall-tau is higher in the observed data also have relatively higher values in the simulated data, for example, in the United Kingdom and Ireland. Capturing higher dependencies is important, as this also influences how frequently co-occurring extremes (where multiple variables are extreme at the same time) are detected.

**Fig. 9:**
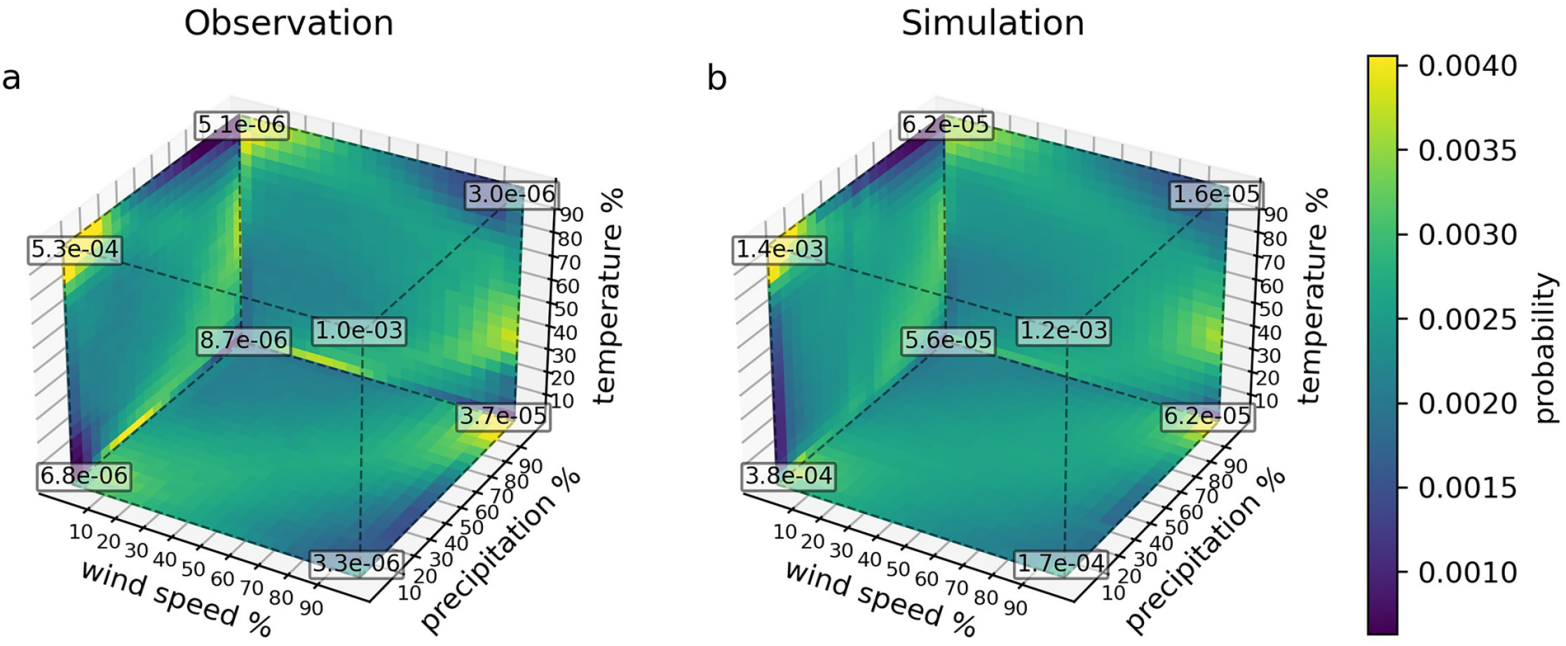
3D heatmaps showing the joint probability distribution of two variables across percentile ranges, averaged over the entire dataset. Each cell indicates the probability that both variables simultaneously fall within the corresponding percentile intervals for. The numbers in the corners illustrate the probability that all three variables are extreme.

Figure 9 shows the probability that a different pair of variables fall within specific percentiles. For example, it indicates the likelihood that both precipitation and wind speed exceed the 95^th^ percentile, as well as the probability that they are within a moderate range, such as between the 50^th^ and 55^th^ percentiles. Here, we see that both the observed and simulated data shows very similar patterns. For instance, there is a higher probability that wind speed and precipitation are low when temperature is high, or when precipitation is high, wind speed is also high. The numbers in each corner of the cubes in Fig. 9 indicate the probability that all three variables are extreme at the same time. For example, the bottom left number is the probability that all variables are below the 5^th^ percentile while the bottom right number is the probability that temperature and precipitation are 5^th^ percentile while wind speed is above the 95^th^ percentile. In both the observed and simulated data, the likelihood of these co-occurring extremes in all three variables are low. However, they are higher in the simulated data, possibly because of the small biases, such as higher wind speeds. The most important aspect is that these co-occurring extremes are represented in the simulated data, indicating that the simulated data captures their occurrence, despite minor biases.

## Usage Notes

The MYRIAD-SIM data are suitable to analyze plausible weather scenarios on a European scale. However, a couple of limitations should be noted. Firstly, the model was fit on data from 2008 to 2023, which means it is predominately representative of the weather in the current climate and is not suitable yet for the evaluation of future climate conditions. Additionally, MYRIAD-SIM was trained using ERA5-land, and therefore, inherently has certain biases of the ERA5-land data. These biases include an underestimation of wind speed and precipitation over high elevation areas^38,39^, and the underestimation of temperature, predominately over urban areas^40,41^.

In order to overcome these biases, MYRIAD-SIM is suitable to be fit using longer periods of observation, as well as climate data input to reflect different climates. MYRIAD-SIM can also be fit to higher resolution local spatiotemporal gridded data to improve accuracy on a local scale. Hence, we plan to apply this approach to case studies in future work.

## Data Availability

The MYRIAD-SIM Python codes are publicly available on GitHub (https://github.com/judithclaassen/MYRIAD-SIM). The *V**i**n**e**C**o**p**u**l**a**s* package which this model is based is also avilable on GitHub (https://github.com/VU-IVM/VineCopulas) and archived on Zenodo^31^.
